# Evaluation of Gonadal Alterations in a Population Environmentally Exposed to a Mixture of Endocrine Active Pesticides

**DOI:** 10.3390/ijerph18052355

**Published:** 2021-02-28

**Authors:** Mar Requena-Mullor, Angeles Navarro-Mena, Ruqiong Wei, Olga López-Guarnido, David Lozano-Paniagua, Raquel Alarcon-Rodriguez

**Affiliations:** 1 Department of Nursing, Physiotherapy and Medicine, University of Almería, 04120 Almería, Spain; mrm047@ual.es (M.R.-M.); dlozano@ual.es (D.L.-P.); ralarcon@ual.es (R.A.-R.); 2 Nursing Department, Torrecardenas Hospital, 04009 Almeria, Spain; angelamena92@gmail.com; 3 Department of Rehabilitation Medicine, First Affiliated Hospital of Guangxi Medical University, Nanning 530021, China; weiruqiongxibanya@163.com; 4 Department of Legal Medicine and Toxicology, Medical School, University of Granada, 18016 Granada, Spain

**Keywords:** pesticide, endocrine disruption, gonadal dysfunction, ovarian cancer, testicular cancer

## Abstract

Although there are studies that show that some pesticides produce gonadal dysfunction and gonadal cancer in different animals, there are not many studiesregardinghumans. This study determined the prevalence and risk in humans of developing ovarian or testicular dysfunction or cancer in areas with distinct exposure to pesticides, which have endocrine disrupting properties. A population-based case-control study was carried out on humans living in ten health districts of Andalusia (Southern Spain) classified as areas of high or low environmental exposure to pesticides according to agronomic criteria. The study population included 5332 cases and 13,606 controls. Data were collected from computerized hospital records between 2000 and 2018.The risk of gonadal dysfunction or cancer was significantly higher in areas with higher use of pesticides in relation to those with lower use.

## 1. Introduction

Pesticides are unique, intrinsically toxic chemicals designed to be widely spread into the environment to kill off pests [1]. Moreover, pesticides produce adverse effects and are contaminants in the areas where they are used. This contamination may affect surface water and groundwater, soil, agricultural products, and plant debris; thus, residential proximity to pesticide-treated farmland is an important pesticide exposure pathway that ultimately has an effect on human health [2].

Pesticides have been in use for decades around the world due to their benefits for agriculture. About 2.7 million tons of pesticides were used worldwide between 2011and 2012, according to the United States Environmental Protection Agency (US-EPA) [3]. Human health, as well as the environment, can be affected if pesticides are not correctly and safely used [4,5]. In fact, exposure to pesticides has turned into an important issue of public health concern throughout the world. Chronic exposure to these xenobiotic compounds could trigger some biochemical alterations on target organs, including gonads, and lead to the appearance of several diseases: this study focuseson gonadal cancer and gonadal dysfunction.

Growing exposure to several different chemicals has caused multiple abnormalities in the reproductive system of human beings and different animal species. According to the definition of the World Health Organization (WHO), an endocrine disrupting chemical (EDC) is an exogenous substance (or mixture of them) that alters the function of the endocrine system and consequently causes adverse effects in an intact organism, its progeny, or their populations [6]. Tons of these chemicals are available on the global market. EDCs are a heterogeneous group of chemicals, including polychlorinated biphenyls (PCBs), polybrominated diethyl ethers (PBDEs), dioxins, plasticizers (bisphenol A (BPA) and phthalates), pesticides (methoxychlor, chlorpyrifos, dichlorodiphenyltrichloroethane (DDT), fungicides (vinclozolin), and herbicides). Exposure to them is very toxic and hazardous to the reproductive system [7]. They act on the hypothalamo–hypophyseal–gonadal axis (HHG) by producing a hormonal alteration that results in a modulation of development and gonadal growth which can lead to ovarian or testicular dysfunction [8].

Hormonal balance, particularly related to sexual hormones, is important in relation to the sensitive human fertility process [9]. Therefore, in human populations, the majority of the studies point towards an association between exposure to EDC and male and/or female reproductive system disorders, such as infertility, endometriosis, poor sperm quality, and/or poor sperm function [10]. Several studies, corroborated by animal research, have reported that the sperm function and sperm quality are more deficient in men who have been exposed to pesticides; these men also have higher rates of infertility [9,11,12,13]. There is not much research into the effects of pesticides on the human ovary, but studies on animals have shown an adverse effect of pesticides on the ovary, resulting in a reduction of ovarian weight, follicle growth, and oocyte viability and/or an increase in atresia [14,15,16].

In recent years, an increase in the incidence of gonadal cancer has been observed. This increase could not only be motivated by an aging population but also by the presenceof carcinogens in the workplace and environment [17]. There are many risk factors studied for cancer, such as obesity, tobacco, alcohol, sedentary lifestyle. Recent studies have shown that exposure to certain environmental pollutants such as pesticides are associated with an increase in the number of cancer cases [18]. Currently, ovarian cancer represents the ninth most significant cause of malignancies in women with an age standardized rate (ARS, per 100,000) of 6.6 [19]. In developed countries, ovarian cancer is the second most common genital tract malignancy, with women having a 1–2% life-time risk of developing the disease [20]. Moreover, it is important to study this type of cancer because it is the most lethal gynecological malignancy with an overall five-year survival of 46% [20]. Regarding testicular cancer, its age standardized rate (ARS, per 100,000) is 1.8 [19].

In addition to the carcinogenic effect of many pesticides, they can also produce other chronic effects on human health, such as neurotoxicity, genotoxicity, mutagenicity, reproductive toxicity, and endocrine disruption, among others [21,22,23,24]. Animal and in vitro studies have supported the hypothesis that EDCs affect the hormone-dependent pathways responsible for male and female gonadal development, either through direct interaction with hormone receptors or via epigenetic and normal cell-cycle regulatory modes of action, such as those observed in certain phthalates inhibiting the antralfollicle growth and stopping cell cycle progression by inhibiting the expression of the cyclins E1, A2, and B1 [10]. Several studies have measured serum levels of pituitary hormones (luteinizing hormone (LH), follicle-stimulating hormone (FSH) and prolactin) and steroid hormones (estradiol (E2), testosterone (T) and progesterone). They found that there were serum variations of these hormones in animals exposed to pesticides compared with those that were not exposed. These variations depended on the dose used and the type of pesticide, such as organophosphate pesticides [25,26,27,28,29] and organochlorine pesticides [11]. There are very few epidemiological studies conducted in the female population, but in men there are many studies that have measured the male hormonal profile and exposure biomarkers [12].

The aim of this study was to assess the association between environmental exposure to pesticides and prevalence of gonadal cancer and gonadal dysfunction.

## 2. Materials and Methods

### 2.1. Design

A population-based case-control study was conducted in selected areas of Andalusia (Southern Spain) with different pesticide exposure because of diverse patterns of pesticide use. The objective was to determine whether there is any association between pesticide exposure and the development of gonadal cancer or gonadal dysfunction.

### 2.2. Criteria for the Selection of the Study Areas and Pesticide Exposure

**Figure 1:**
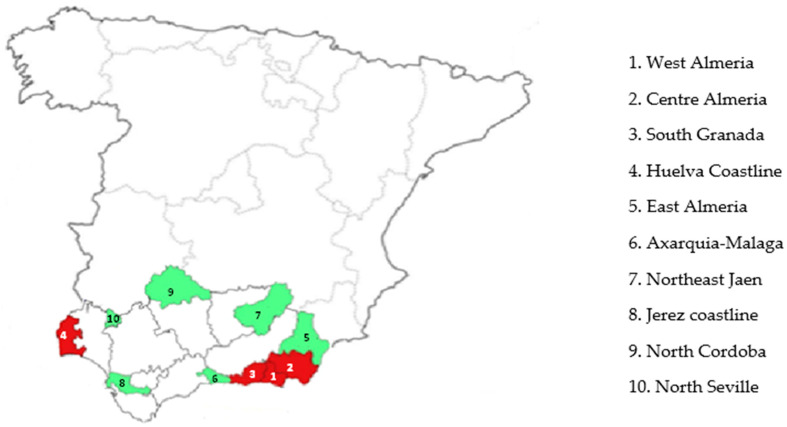
Geographic distribution of the study areas: high use of pesticides (dark red) and low use of pesticides (clear green).

The selected areas in Andalusia (Southern Spain) were classified into two groups (high versus low use of pesticides) according to agronomic criteria (tons of pesticide used and land area dedicated to intensive agriculture in greenhouses covered with plastic). Areas of high pesticide use included West Almeria, Centre of Almeria, South Granada, and Huelva Coastline. Conversely, areas of low pesticide use were comprised by Axarquia (Malaga), Jerez coastline (Cadiz), East Almeria, Northeast Jaen, North Cordoba, and North Seville (Figure 1).

The consumption of pesticides in areas considered as high exposure was 14,002.1tons (91.4%; 40.47 Tm/km^2^). These areas occupied 93.2% of the total surface of greenhouses in Andalusia (period 2016–2017). By contrast, in low exposure areas 1305.7 tons (8.6%, 37.19 Tm/km^2^) of pesticides were used and occupied only 6.8% of the total surface of greenhouses in Andalusia [30].

According to previous studies and information provided by the agronomists working in the area over the study period, the most often used insecticides were macrocyclic lactones (abamectin, spinosad), neonicotinoids (imidacloprid, acetamiprid), pyrethroids (cypermethrin, deltamethrin), and others (indoxacarb, azadirachtin, spiromesifen, and Bacillus thuringiensis). The most frequently used fungicides included: triazoles (tebuconazol, triadimenol, miclobutanil), anilino-pyrimidines (cyprodinil, mepanipyrim, pyrimethanil), copper salts (copper oxychloride), and others (phenyl pyrrole, thiophanate methyl, fluopicolide, chlorthalonil, propamocarb, dimethomorph, azoxystrobin) [31].

### 2.3. Study Population and Gonadal Diseases

A total of 5332 individuals diagnosed with any gonadal disease between January 2000 and December 2018 were enrolled in this study. According to the level of exposure to pesticides these cases were divided into two groups: the high-exposure-area group (consisting of 2975 individuals living in areas with a widespread use of pesticides) and the low-exposure-area group, which consisted of 2357 cases. The control population involved 13,606 individuals without gonadal disease (6647 from areas of high pesticide use and 6959 from areas of low pesticide use), recruited from the Primary Care Services from areas of exposure. To minimize any difference in the background exposure to pesticides, cases and controls lived in the same area and were matched by age and sex.

Cases were collected from computerized records of the Basic Minimum Data Set (BMDS) for the Public Health Service of Andalusia over a period of 19 years (January 2000 to December 2018). The BMDS Andalusia is an administrative record that contains a set of clinical, demographic, and administrative variables that summarizes what happened to users before an episode of hospital care. It provides basic information about the users, the center and the unit that attends them, and about their care process.

The ninth revision of the International Classification of Diseases (ICD-9) was used to define the diagnosis of gonadal diseases [32]: ovarian cancer (183), testicular cancer (186), ovarian dysfunction (256), and testicular dysfunction (257).

Exclusion criteria were: (1) participants <18 years; (2) to live in geographical areas not selected for the study.

### 2.4. Statistical Analysis

The data were analyzed using the SPSS statistical software package (SPSS 25.0 for Windows, IBM, Armonk, NY, USA). Frequencies and percentages were calculated for categorical variables; and means and standard deviations for quantitative variables (e.g., age). In addition, the prevalence rate and risk of developing gonadal disease were calculated in areas of high and low pesticide use using the Chi-squared test. Odds ratio (OR) and their corresponding 95% confidence interval (95% CI) were also calculated. The Student’s T test was used to compare age differences in the population between the two study areas. TheKolmogorov–Smirnov test was used for testing normality.

Multiple binary logistic regression was carried out to assess the risk of having gonadal disease adjusted for age, gender, and areas of pesticide use as a surrogate of exposure, as these were considered to have an influence on the statistical model. The level of statistical significance was established for a value of *p* < 0.05.

## 3. Results

The average age of the individuals diagnosed with ovarian cancer was 51.97 ± 15.68 years, for testicular cancer it was 31.17 ± 17.04 years, for ovarian dysfunction it was 33.57 ± 9.40 years, and for testicular dysfunction it was 43.16±19.17 years, in the areas of high pesticide use, versus 53.54 ± 16.15 years for ovarian cancer, 31.90 ± 17.45 years for testicular cancer, 33.93 ± 11.86 years for ovarian dysfunction, and 43.93 ± 15.96 years for testicular dysfunction, in the areas of low pesticide use. When comparing the age of individuals between the two study areas for each of the gonadal diseases studied, no significant differences were found except for ovarian cancer in which higher age can be observed for those individuals located in the low exposure area (Table 1).

**Figure 2:**
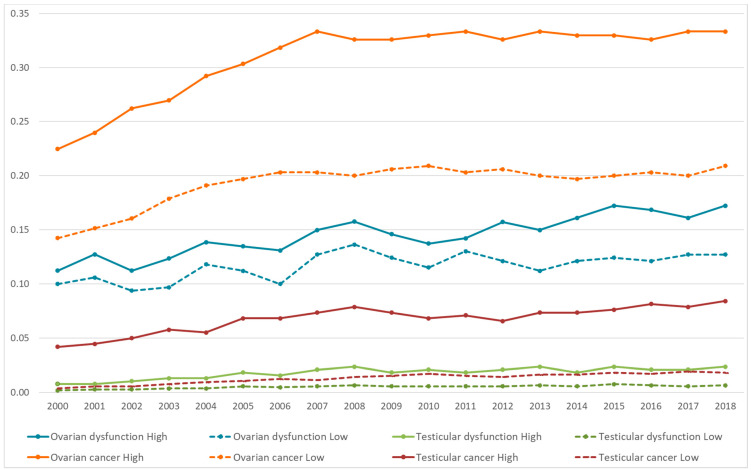
Distribution of the gonadal diseases (prevalence per 1000 inhabitants) as a whole and stratified by the geographical areas studied (high and low pesticide exposure).

Figure 2 shows the trend for the adjusted prevalence rates per 1000 inhabitants separately for each gonadal disease, stratified by areas of high and low pesticide use, over the 19 years of study (2000–2018). The temporospatial pattern of the gonadal diseases studied shows a parallel trend for ovarian cancer, testicular cancer, and testicular dysfunction, but not for ovarian dysfunction, whose prevalence showed variations throughout the period for the areas of high and the areas of low pesticide use.

Significantly increased prevalence rates for ovarian cancer and testicular cancer were observed in areas of greater pesticide use than in those of lesser use. A slightly but significantly greater prevalence rate was also observed for ovarian dysfunction and testicular dysfunction in the areas of high relative to low pesticide use (Table 2).

Table 2 also shows the risk of having the diverse gonadal diseases studied, expressed as odds ratios (OR), for areas of high relative to low pesticide use. A significant increased risk of ovarian cancer, testicular cancer, ovarian dysfunction, and testicular dysfunction was found in the areas of high versus low pesticide use. The highest risks were observed for ovarian cancer and testicular cancer, with OR of 1.39 and 1.37, respectively, followed by testicular dysfunction and ovarian dysfunction (overall ORs of 1.36, and 1.12, respectively). These differences were statistically significant in both cancer and gonadal dysfunction.

The results obtained from the multiple logistic regression analysis (adjusted for age, sex, and areas of high versus low use of pesticides) showed that people residing in areas of high use of pesticides showed a significantly higher risk of having the studied diseases. Environmental pesticide exposure was associated with an increased risk of gonadal diseases, with the order of higher to lower risks being observed for testicular cancer, ovarian cancer, ovarian dysfunction, and testicular dysfunction. Regarding age, the gonadal diseases studied were positively associated with age, with an increased risk ranging from 0.97% for ovarian dysfunction to 1.10% for testicular dysfunction (Table 3).

## 4. Discussion

The present study tries to assess whether living in areas with long-term pesticide exposure, as a result of greater use of these compounds in intensive agriculture, is associated with an increased prevalence and risk of gonadal alterations. Pesticides are a group of chemicals used in crops and residential pest control. Due to their widespread use, residues of pesticides can be foundin food or drinking water [33] and the general population could be exposed to them from occupational and environmental sources [34,35]. This issue is a concern for the main health authorities. Andalusia has special geographical and climatological characteristics in terms of the development of intensive agricultural activities and the distribution of greenhouses, which require the continuous use of pesticides. The population residing near areas with intensive agriculture will be exposed to the effects of pesticides, as are agricultural workers [36].

Although current scientific evidence in humans on this subject is scarce, the existing literature suggests that exposure to pesticides is associated with changes in gonadal function, such as genital infertility, poor sperm quality or changes in sex hormone levels, malformations, endometriosis, testicular cancer or ovarian cancer [10,31,37].

### 4.1. Pesticide Exposure and Gonadal Dysfunction

The multivariate analysis performed in this study revealed that women who lived in areas of high environmental exposure to pesticideshad 1.38 times greater odds of suffering from ovarian dysfunction than those who lived in areas of low exposure. Likewise, men who lived in areas of high environmental exposure to pesticides had 1.29 times greater risk of suffering from testicular dysfunction than those who lived in areas of low exposure (Table 3). These results could be due to the fact that pesticides can be bioaccumulative [25,38] and because the prevalence of the disease typically increases with age. The age of the population in the two study areas were similar with no statistically significant differences.

Endocrine disruptors, including pesticides, modify endocrine properties in animals and humans by either mimicking or blocking endocrine actions. Endocrine disruptors can interfere with receptor binding, steroidogenesis, and metabolism of hormones. In fact, pesticides with endocrine-disrupting activity have estrogenic and antiandrogenic activities that can alter the sex-steroid synthesizing enzymes [15].

Although hormonal levels and sperm quality have not been measured directly in the present study, our results indicate that the prevalence and odds of having gonadal dysfunction is higher among populations living in areas withhigh pesticide use than those living in areas with low pesticide use (Table 2). Exposure to certain pesticides can affect the sperm motility, reducing it [12], and spermatogenesis will also be affected, either directly by damaging or destroying Sertoli cells, or indirectly by altering hormonal signaling [25]. A decrease in ejaculation volume, low sperm count, impaired motility sperm, and disruption of reproductive hormone levels in men exposed to the endocrine disruptors was reported in the population with environmental exposure to organochlorine and organophosphate pesticides [12,39].

Additionally, pesticides as well as other endocrine disruptors are capable of interfering with the female reproductive system by affecting the structure and function of the reproductive organs, including the ovary, uterus, vagina, and anterior pituitary. Unfortunately, the results of studies that aim to explain why pesticides affect the female reproductive health and their mechanisms are limited and inconsistent. However, it is known that pesticides and other EDCs are able to inhibit follicle growth or to increase atresia and apoptosis in animal models, and disrupt steroid hormone levels in animals and women [40].

### 4.2. Pesticide Exposure and Gonadal Cancer

The findings from this study showeda significantly higher prevalence and risk of developing gonadal cancer(both testicularcancer in men and ovarian cancer in women)in areas of high pesticide use compared to those of low pesticide use (Table 2 and Table 3). The risk factors for testicular cancer are not well understood, but presume prior cryptorchidism, prior unilateral testicular cancer, and a family history of testicular cancer [41]. Prenatal exposure to pesticides may lead to testicular dysgenesis syndrome (TDS) in adult life, a kind of reproductive abnormality that includes cryptorchidism, hypospadias, poor semen quality, and a predisposition to testicular cancer. Some studies have considered that the origin of TDS is the inhibition of the action of androgens in fetal development that leads to dysfunction of Sertoli and Leydig cells [42].The number of studies that have been performed in recent years has been increasing, which highlightsthe growing interest that this topic represents for health authorities and for the scientific world, especially in developed countries [10].

The best part of the articles, as occurred in ovarian cancer studies, were carried out on organochlorine pesticides. Most of them exhibited a statistically significant increase in the incidence of this kind of cancer with the exposure to pesticides in the occupational ambiance [43,44,45,46]. A complete review carried out in 2012 suggested that the majorityof the studies performed in the last decades showed a link between employment in the agricultural sector and an increased risk of testicular cancer [41]. They also showed that only dichlorodiphenyldichloroethylene and chlordanes were found to be associated with an increased risk of testicular cancer. However, they did not report an association between testicular cancer and other organochlorine pesticides, including dichloro-diphenyl-trichloroethane, dieldrin, mirex, hexachlorobenzen, hexachlorocyclobenzene, etc. In addition, another study found a significant association between testicular cancer and household insecticide use [44].

Parrón et al. (2014) observed a 91% higher risk of testicular cancer in the population with high pesticide exposure [18]. These findings support the hypothesis that the increase in the incidence of testicular cancer in recent decades could be related, at least in part, to the accumulation of some pesticides in the environment [18,44].

Regarding ovarian cancer in women, not many significant studies have been found. The research conducted on pesticide exposure (and other endocrine disruptors) has largely focused on breast, endometrial, prostate, and testicular cancers, whereas limited work has been done with respect to ovarian cancer [47]. Although it is still debated if ovarian cancer is estrogen-dependent, data from experiments conducted in vitro showed that endocrine disruptors stimulated the proliferation of positive BG-1 ovarian cancer cells via the estrogen signaling pathway [48]. In this sense, Lerro et al. observed an increased risk with organophosphate insecticide use, especially diazinon, for several hormonally mediated cancers, including ovary cancer [49]. It is well known that this kind of pesticides have shown to exhibit estrogenic properties, and to have a genotoxic effect, so they could have the potential to cause hormonally mediated effects in relation to cancer risk among women.

Epigenetics has studied some elements regarding cancer detection and cancer biology, but few works have been found to address the effect that certain endocrine disruptors have on epigenetic modifications in hormone receptor genes. The formation of some ovarian tumors could be motivated by these epigenetic modifications [47]. In this sense, a study carried out in Fischer inbred rats has shown a hypermathylation of the ERβ promoter sequences caused by the exposure to the organochlorine pesticide methoxychlor [50].

In addition, one might speculate on the potential role of the indirect actions of endocrine disruptors on ovarian cancer risk, especially disruptors that possess anti-estrogenic effects in the hypothalamus, which lead to an increased production of gonadotropins. Since gonadotropins directly stimulate the gonads, these endocrine disruptors may also impact the risk of ovarian cancer development [48].

Oxidative stress and the immune system are also a considerable influence since exposure to pesticides could induce reactive oxygen species (ROS) generation and secrete different pro-inflammatory cytokines that lead to chronic inflammatory reactions and forma tumor-promoting microenvironment, showing significant DNA damage [51]. Hence, exposure to pesticides could trigger an oxidative stress environment in which tumor-associated macrophages release different pro-inflammatory cytokines. These released cytokines can produce low-intensity systemic inflammation that, along with oxidative molecules produced by the oxidative stress ambiance, can lead to alterations conducting to the appearance and subsequent progression of ovarian cancer.

### 4.3. Limitations and Strengths of This Study

The ecological nature of the exposure data is the major limitation of the study. While specific data on frequency and duration of exposure to pesticides were not available at the individual level, an aggregated measure of exposure was used which allowed us to make comparisons between people living in areas classified as high and low pesticide use based on quantitative agronomic criteria. This methodology enabled us to calculate the risk of developing gonadal diseases.

Another limitation is the difficulty in determining which pesticides are responsible for the disrupting gonadal effect as farmers generally apply several pesticides for their crops either concurrently or sequentially. However, this study provides an approach to a real-world exposure scenario to mixtures of pesticides. Additionally, the potential contribution of co-exposure to other chemicals that might be present at higher concentrations in the area of greater pesticide use cannot be excluded. However, no evidence for this possibility has been found.

Conversely, the major strength of this study is the large number of cases, which amounted to 5332 individuals diagnosed with diverse gonadal diseases (ovarian cancer, testicular cancer, ovarian dysfunction, testicular dysfunction). Furthermore, this study allows for examining the temporal trend for those disorders over a period of 19 years.

## 5. Conclusions

This study found an association between environmental pesticide exposure (resulting from a higher pesticide use in areas of intensive agriculture under plastic greenhouses) and an increased risk of gonadal diseases. The risk for ovarian cancer, testicular cancer, ovarian dysfunction, and testicular dysfunction was higher for the population living in the areas with a higher use of pesticides. The results of this study support and extend previous findings indicating that the risk of gonadal dysfunction and gonadal cancer increases in human populations living in areas of high exposure to pesticides. Thus, environmental pesticide exposure (or concurrent exposures to multiple pesticides) may be a contributing risk factor for developing gonadal diseases. However, the limitations inherent to the study design warrant further research to ascertain whether there exists a true causal association.

## Figures and Tables

**Table 1:** Comparison of the mean age by the geographical areas studied (high and low pesticide exposure).

Gonadal Alterations	Exposure	Age (Mean (SD))	*p* Value *
OvarianCancer	High exposure	51.97 (15.68)	<0.05
	Lowexposure	53.54 (16.15)	
Testicular Cancer	High exposure	31.17 (17.04)	>0.05
	Lowexposure	31.90 (17.45)	
OvarianDysfunction	High exposure	33.57 (9.40)	>0.05
	Lowexposure	33.93 (11.86)	
Testicular Dysfunction	High exposure	43.16 (19.17)	>0.05
	Lowexposure	43.93(15.96)	

* Student’s *T* test.

**Table 2:** Prevalence (rate per 1000 inhabitants), odds ratio (OR), and 95% confidence interval (95% CI) for gonadal diseases in the population living in areas of high pesticide exposure relative to areas of low exposure.

Gonadal Alterations (n = 5332)	High Exposure	Low Exposure	OR (95% CI)	*p* Value *
OvarianCancer (n = 2838)	4.87	3.04	1.39 (1.15–1.38)	<0.001
Testicular Cancer (n = 766)	1.04	0.19	1.37 (1.12–1.60)	<0.001
OvarianDysfunction (n = 1489)	2.22	1.83	1.12 (1.10–1.30)	<0.05
Testicular Dysfunction (n = 239)	0.27	0.08	1.36 (1.02–1.89)	<0.05

* Pearson’s Chi-squared test.

**Table 3:** Stepwise multiple logistic regression analysis of gonadal alterations adjusted for exposure to pesticides and age.

Thyroid Alterations	Risk Factor	OR *	95% CI	*p* Value
OvarianCancer	Exposure	1.41	1.24–1.60	<0.001
	Age	1.03	1.03–1.04	<0.001
Testicular Cancer	Exposure	1.59	1.37–1.85	<0.001
	Age	1.01	1.07–1.16	<0.001
OvarianDysfunction	Exposure	1.38	1.05–1.82	<0.01
	Age	0.97	0.96–0.97	<0.001
Testicular Dysfunction	Exposure	1.29	1.13–1.34	<0.01
	Age	1.10	1.09–1.18	<0.05

* Models were adjusted for the following variables: age, environmental pesticide exposure (1: areas of high pesticide use; 0: areas of low pesticide use).

## Data Availability

The data presented in this study are available on request from the authors. The data are not publicly available due to privacy or ethical restrictions.

## References

[1] Hernández A.F., Parrón T., Tsatsakis A.M., Requena M., Alarcón R., López-Guarnido O. (2013). Toxic effects of pesticide mixtures at a molecular level: Their relevance to human health. Toxicology.

[2] Becerra T.A., Bravo L.X. (2010). La agricultura intensiva del poniente almeriense. Diagnóstico e instrumentos de gestión ambiental. M + A. Rev. Electrónica Medioambiente.

[3] Atwood D., Paisley-Jones C. (2017). Pesticides Industry Sales and Usage. 2008–2012 Market Estimates.

[4] Jabłońska-Trypuć A., Wołejko E., Wydro U., Butarewicz A. (2017). The impact of pesticides on oxidative stress level in human organism and their activity as an endocrine disruptor. J. Environ. Sci. Heal. Part B Pestic. Food Contam. Agric. Wastes.

[5] Pimentel D. (1996). Green revolution agriculture and chemical hazards. Sci. Total Environ..

[6] Damstra T., Barlow S., Bergman A., Kavlock R.J., Der Kraak G.V. (2002). Global Assessment of the State-of-the-Science of Endocrine Disruptors.

[7] Piazza M.J., Urbanetz A.A. (2019). Environmental toxins and the impact of other endocrine disrupting chemicals in women’s reproductive health. J. Bras. Reprod. Assist..

[8] Senthilkumaran B. (2015). Pesticide- and sex steroid analogue-induced endocrine disruption differentially targets hypothalamo-hypophyseal-gonadal system during gametogenesis in teleosts—A review. Gen. Comp. Endocrinol..

[9] Neghab M., Moemenbellah-Fard M.D., Naziaghdam R., Salahshour N., Kazemi M., Alipour H. (2014). The effects of exposure to pesticides on the fecundity status of farm workers resident in a rural region of Fars province, southern Iran. Asian Pac. J. Trop. Biomed..

[10] Sifakis S., Androutsopoulos V.P., Tsatsakis A.M., Spandidos D.A. (2017). Human exposure to endocrine disrupting chemicals: Effects on the male and female reproductive systems. Environ. Toxicol. Pharmacol..

[11] Da Cuña R.H., Pandolfi M., Genovese G., Piazza Y., Ansaldo M., Lo Nostro F.L. (2013). Endocrine disruptive potential of endosulfan on the reproductive axis of Cichlasoma dimerus (Perciformes, Cichlidae). Aquat. Toxicol..

[12] Melgarejo M., Mendiola J., Koch H.M., Moñino-García M., Noguera-Velasco J.A., Torres-Cantero A.M. (2015). Associations between urinary organophosphate pesticide metabolite levels and reproductive parameters in men from an infertility clinic. Environ. Res..

[13] Chen X., Wang J., Zhu H., Ding J., Peng Y. (2015). Proteomics analysis of Xenopus laevis gonad tissue following chronic exposure to atrazine. Environ. Toxicol. Chem..

[14] Monteiro M.S., Pavlaki M., Faustino A., Rêma A., Franchi M., Gediel L., Loureiro S., Domingues I., von Osten R.J., Soares M.V.M.A. (2015). Endocrine disruption effects of p,p’-DDE on juvenile zebrafish. J. Appl. Toxicol..

[15] Rattan S., Zhou C., Chiang C., Mahalingam S., Brehm E., Flaws J.A. (2017). Exposure to endocrine disruptors during adulthood: Consequences for female fertility. J. Endocrinol..

[16] Zhu L.Z., Qi S.Z., Cao F.J., Mu X.Y., Yang Y., Wang C. (2017). Quizalofop-P-ethyl exposure increases estrogen axis activity in male and slightly decreases estrogen axis activity in female zebrafish (Danio rerio). Aquat. Toxicol..

[17] Tebourbi O., Sakly M., Ben K. (2011). Molecular mechanisms of pesticide toxicity. Pesticides in the Modern World—Pests Control and Pesticides Exposure and Toxicity Assessment.

[18] Parrón T., Requena M., Hernández A.F., Alarcón R. (2014). Environmental exposure to pesticides and cancer risk in multiple human organ systems. Toxicol. Lett..

[19] Ferlay J., Colombet M., Soerjomataram I., Mathers C., Parkin D.M., Piñeros M., Znaor A., Bray F. (2019). Estimating the global cancer incidence and mortality in 2018: GLOBOCAN sources and methods. Int. J. Cancer.

[20] Campbell S., Gentry-Maharaj A. (2018). The role of transvaginal ultrasound in screening for ovarian cancer. Climacteric.

[21] Znaor A., Lortet-Tieulent J., Jemal A., Bray F. (2014). International variations and trends in testicular cancer incidence and mortality. Eur. Urol..

[22] Albers P., Albrecht W., Algaba F., Bokemeyer C., Cohn-Cedermark G., Fizazi K., Horwich A., Laguna M.P., Nicolai N., Oldenburg J. (2015). Guidelines on testicular cancer: 2015 update. Eur. Urol..

[23] Dabrowski J.M., Shadung J.M., Wepener V. (2014). Prioritizing agricultural pesticides used in South Africa based on their environmental mobility and potential human health effects. Environ. Int..

[24] Sugeng A.J., Beamer P.I., Lutz E.A., Rosales C.B. (2013). Hazard-ranking of agricultural pesticides for chronic health effects in Yuma County, Arizona. Sci. Total Environ..

[25] Cremonese C., Piccoli C., Pasqualotto F., Clapauch R., Koifman R.J., Koifman S., Freire C. (2017). Occupational exposure to pesticides, reproductive hormone levels and sperm quality in young Brazilian men. Reprod. Toxicol..

[26] Aguilar-Garduño C., Lacasaña M., Blanco-Muñoz J., Rodríguez-Barranco M., Hernández A.F., Bassol S., González-Alzaga B., Cebrián M.E. (2013). Changes in male hormone profile after occupational organophosphate exposure. A longitudinal study. Toxicology.

[27] Ventura C., Nieto M.R.R., Bourguignon N., Lux-Lantos V., Rodriguez H., Cao G., Randi A., Cocca C., Núñez M. (2016). Pesticide chlorpyrifos acts as an endocrine disruptor in adult rats causing changes in mammary gland and hormonal balance. J. Steroid Biochem. Mol. Biol..

[28] Skolness S.Y., Blanksma C.A., Cavallin J.E., Churchill J.J., Durhan E.J., Jensen K.M., Johnson R.D., Kahl M.D., Makynen E.A., Villeneuve D.L. (2013). Propiconazole inhibits Steroidogenesis and Reproduction in the Fathead Minnow (pimephales promelas). Toxicol. Sci..

[29] Joshi S.C., Mathur R., Gulati N. (2007). Testicular toxicity of chlorpyrifos (an organophosphate pesticide) in albino rat. Toxicol. Ind. Health.

[30] Consejería de Agricultura, Pesca y Desarrollo Rural, Junta de Andalucía (2017). Cartografía de Invernaderos en Almería, Granada y Málaga; Año. https://www.juntadeandalucia.es/export/drupaljda/Cartografia%20_inv_AL_GR_MA_SEE.pdf.

[31] García-García C.R., Parrón T., Requena M., Alarcón R., Tsatsakis A.M., Hernández A.F. (2016). Occupational pesticide exposure and adverse health effects at the clinical, hematological and biochemical level. Life Sci..

[32] (1978). International Classification of Diseases: [9th] Ninth Revision, Basic Tabulation List with Alphabetic Index.

[33] European Food Safety Authority (2014). The 2011 European Union report on pesticide residues in food. EFSA J..

[34] Mantovani A. (2016). Endocrine disrupters and the safety of food chains. Horm. Res. Paediatr..

[35] Fantke P., Charles R., de Alencastro L.F., Friedrich R., Jolliet O. (2011). Plant uptake of pesticides and human health: Dynamic modeling of residues in wheat and ingestion intake. Chemosphere.

[36] Requena M., Parrón T., Navarro A., García J., Ventura M.I., Hernández A.F., Alarcón R. (2018). Association between environmental exposure to pesticides and epilepsy. Neurotoxicology.

[37] Warembourg C., Debost-Legrand A., Bonvallot N., Massart C., Garlantézec R., Monfort C., Gaudreau E., Chevrier C., Cordier S. (2016). Exposure of pregnant women to persistent organic pollutants and cord sex hormone levels. Hum. Reprod..

[38] Kjeldsen L.S., Ghisari M., Bonefeld-Jørgensen E.C. (2013). Currently used pesticides and their mixtures affect the function of sex hormone receptors and aromatase enzyme activity. Toxicol. Appl. Pharmacol..

[39] Riana Bornman M., Bouwman H. (2012). Environmental pollutants and diseases of sexual development in humans and wildlife in South Africa: Harbingers of impact on overall health?. Reprod. Domest. Anim..

[40] Gore A.C., Chappell V.A., Fenton S.E., Flaws J.A., Nadal A., Prins G.S., Toppari J., Zoeller R.T. (2015). Executive summary to EDC-2: The Endocrine Society’s second Scientific Statement on endocrine-disrupting chemicals. Endocr. Rev..

[41] McGlynn K.A., Trabert B. (2012). Adolescent and adult risk factors for testicular cancer. Nat. Rev. Urol..

[42] Sharma A., Mollier J., Brocklesby R.W.K., Caves C., Jayasena C.N., Minhas S. (2020). Endocrine-disrupting chemicals and male reproductive health. Reprod. Med. Biol..

[43] Paoli D., Giannandrea F., Gallo M., Turci R., Cattaruzza M.S., Lombardo F., Lenzi A., Gandini L. (2015). Exposure to polychlorinated biphenyls and hexachlorobenzene, semen quality and testicular cancer risk. J. Endocrinol. Investig..

[44] Giannandrea F., Gandini L., Paoli D., Turci R., Figà-Talamanca I. (2011). Pesticide exposure and serum organochlorine residuals among testicular cancer patients and healthy controls. J. Environ. Sci. Health. B.

[45] Frost G., Brown T., Harding A.H. (2011). Mortality and cancer incidence among British agricultural pesticide users. Occup. Med..

[46] Lerro C.C., Koutros S., Andreotti G., Sandler D.P., Lynch C.F., Louis L.M., Blair A., Parks C.G., Shrestha S., Lubin J.H. (2019). Cancer incidence in the Agricultural Health Study after 20 years of follow-up. Cancer Causes Control.

[47] Samtani R., Sharma N., Garg D. (2018). Effects of endocrine-disrupting chemicals and epigenetic modifications in ovarian cancer: A review. Reprod. Sci..

[48] Rachoń D. (2015). Endocrine disrupting chemicals (EDCs) and female cancer: Informing the patients. Rev. Endocr. Metab. Disord..

[49] Lerro C.C., Koutros S., Andreotti G., Friesen M.C., Alavanja M.C., Blair A., Hoppin J.A., Sandler D.P., Lubin J.H., Ma X. (2015). Organophosphate insecticide use and cancer incidence among spouses of pesticide applicators in the Agricultural Health Study. Occup. Environ. Med..

[50] Zama A.M., Uzumcu M. (2009). Fetal and neonatal exposure to the endocrine disruptor methoxychlor causes epigenetic alterations in adult ovarian genes. Endocrinology.

[51] Shah H.K., Sharma T., Banerjee B.D. (2020). Organochlorine pesticides induce inflammation, ROS production, and DNA damage in human epithelial ovary cells: An In Vitro study. Chemosphere.

